# Physician Payments from Industry Are Associated with Greater Medicare Part D Prescribing Costs

**DOI:** 10.1371/journal.pone.0155474

**Published:** 2016-05-16

**Authors:** Roy H. Perlis, Clifford S. Perlis

**Affiliations:** 1 Department of Psychiatry, Center for Experimental Drugs and Diagnostics, Massachusetts General Hospital, Boston, Massachusetts, United States of America; 2 Department of Dermatology, Temple University Health System, Philadelphia, Pennsylvania, United States of America; York University, CANADA

## Abstract

**Background:**

The U.S. Physician Payments Sunshine Act mandates the reporting of payments or items of value received by physicians from drug, medical device, and biological agent manufacturers. The impact of these payments on physician prescribing has not been examined at large scale.

**Methods:**

We linked public Medicare Part D prescribing data and Sunshine Act data for 2013. Physician payments were examined descriptively within specialties, and then for association with prescribing costs and patterns using regression models. Models were adjusted for potential physician-level confounding features, including sex, geographic region, and practice size.

**Results:**

Among 725,169 individuals with Medicare prescribing data, 341,644 had documented payments in the OPP data (47.1%). Among all physicians receiving funds, mean payment was $1750 (SD $28336); median was $138 (IQR $48-$394). Across the 12 specialties examined, a dose-response relationship was observed in which greater payments were associated with greater prescribing costs per patient. In adjusted regression models, being in the top quintile of payment receipt was associated with incremental prescribing cost per patient ranging from $27 (general surgery) to $2931 (neurology). Similar associations were observed with proportion of branded prescriptions written.

**Conclusions:**

While distribution and amount of payments differed widely across medical specialties, for each of the 12 specialties examined the receipt of payments was associated with greater prescribing costs per patient, and greater proportion of branded medication prescribing. We cannot infer a causal relationship, but interventions aimed at those physicians receiving the most payments may present an opportunity to address prescribing costs in the US.

## Introduction

Beginning in 2013, the Physician Financial Transparency Reports, or Sunshine Act, mandated that companies manufacturing drugs, devices, and biological agents report individual payments of greater than $10, or $100 in aggregate annually, provided to US physicians. This law followed a 2008 Institute of Medicine (IOM) report describing extensive, though often non-public, commercial conflicts of interest within medicine.[1] One study found that 83% of US physicians received gifts from industry and 28% accepted payments for professional services from industry.[2] The IOM report further described the potential for financial conflicts of interest to inappropriately influence physician practice, a position supported by social sciences research.[3]

Several studies confirm that increased interaction with pharmaceutical representatives influences prescribing behavior. Specifically, increased interaction with industry representatives leads to a greater number of prescriptions for the promoted medications[4]; more generally, in a systematic review, 38/51 studies found that exposure to pharmaceutical company-provided information was associated with increased prescribing.[5] Furthermore, a recent review identified three observational studies showing that policies restricting certain types of interactions between physicians and pharmaceutical representatives yielded an increase in the number of generic, non-promoted, or “first-line” prescriptions depending on the specific study.[6]

While existing evidence suggests effects of physician-pharmaceutical representative interaction overall, important questions remain about the extent and degree of influence that direct financial conflicts of interest exert on physician prescribing behavior, even as efforts continue to expand the Sunshine Act.[7] The availability of Sunshine Act data in conjunction with 2013 Medicare Part D clinician-level summary data afforded an opportunity for direct measurement of the extent and moderators of these associations across a range of specialties.

## Materials and Methods

### Data extraction and cleaning

We accessed the 2013 Medicare Part D clinician-level summary data, which includes information on 99.91% of total prescription claims encompassing prescribers with a valid NPI submitted by the Part D plan sponsors during 2013, and with at least 11 claims.

We also accessed the 2013 Open Payment Program (OPP) general payment data spanning July-December 2013, whose collection is mandated by the Sunshine Act, capturing honoraria, consulting fees, and items of value including food and travel, but not research costs or equity stakes. Notably, while detailed information about each physician's identity and location is collected, including the national provider identifier (NPI), the NPI is explicitly forbidden by statute from release by federal agencies.[8] Therefore, we utilized a string-matching algorithm to associate each physician in the payment data with the best match among the Medicare data. After normalizing all proper names, matching was done with three degrees of stringency: first, by requiring match of last name, first name, and middle initial as well as zip code; second, by requiring match of first and last as well as zip code; and finally by requiring match of first, last, and middle initial. In this fashion we optimized matching while maintaining specificity. Alternative strategies with greater stringency (allowing only full matches in all four fields) or lesser stringency (utilizing fuzzy matching algorithms with variable edit distances) did not yield meaningfully different results and are not presented here.

From the MPD data set, there were 725,169 individuals with MD (Doctor of Medicine) or DO (Doctor of Osteopathic Medicine) degrees or equivalent. From the OPP data set, there were 407,220 individuals with MD or DO degrees. In total, 64,958 individuals could not be reliably matched from the MPD data set; an additional 618 individuals matched to multiple NPIs were identified, yielding 341,644 individuals in both the MPD and OPP data sets (S1 Fig). Remaining individuals in the MPD data set (n = 379,035) were presumed to have received no payments in Q3/4 2013. (Among unmatched OPP individuals, 98% were present in the NPI data set,—i.e., were recognized clinicians without Medicare Part D prescribing data.[9] Manual curation of the unmatched rows from the OPP data set indicated that nearly all were not individuals (eg, they represented LLC's or other corporate entities). Unmatched total OPP payments were significantly smaller than for matched payments—mean $1417.20 (SE 74.6) versus $1749 (SE 48.5); t = 2.87, p<0.01; median $59.06 [IQR 20.68–138.07] versus $137.67 [IQR 48.17–393.76]; Wilcoxon z = 122, p<0.01.

The following physician or practice characteristics were extracted from the MPD data: sex; clinical specialty; zip code/region; number of beneficiaries and claims; proportion of beneficiaries 65 or older; proportion of beneficiaries with low-income subsidy; number of beneficiaries with Prescription Drug Plans (PDP) and Medicare Advantage Prescription Drug (MAPD) plans. We used 2013 US census data to impute median household income in physician zip code, as an approximate index of patient income level. Medical specialties were categorized based on free text to identify those with at least 4,000 physicians; these included psychiatry, neurology, urology, cardiology, dermatology, endocrinology, gastroenterology, hematology/oncology, family or general medicine, and internal medicine. General surgery was also included as a further comparison group.

### Analysis

The primary outcome of interest was cost of prescriptions per beneficiary; secondary outcomes of interest included proportion of branded versus generic prescriptions. We used linear regression to examine association between payment receipt and prescribing measures, with adjustment for potential confounding features of clinician practice. The 95% confidence intervals for beta coefficient for each payment quintile, compared to the reference condition (no payment), was calculated. To investigate linear trend as evidence for dose response, we used a test for contrast between adjacent ordered categories—i.e., to what extent does each quintile differ from the preceding one—based on marginal linear predictions from the regression model as implemented in Stata 13.1 (College Station, TX).[10, 11]

For payment amounts, in light of the highly skewed distribution (S2 Fig, S1B Table)—that is, a small number of individuals receiving particularly large payments, such that the mean payments exceed the median in all cases—we elected a priori to examine 6 categories: no payment, and then each payment quintile. This transformation was selected over alternatives (e.g., logarithmic) to maximize interpretability of results. Individuals in the highest payment quintile were contrasted with all others using Chi Square or T-test, respectively (all differences were significant at p<0.001, so are not presented in tables).

While total cost and claim counts are available for all prescribers, any patient counts with fewer than 11 individuals are redacted to diminish risk of patient re-identification. Under conditions of a truncated or censored independent variable, OLS regression may introduce bias relying on complete case data may likewise introduce bias.[12] For ease of interpretability primary analysis used complete cases, but sensitivity analysis used multiple imputations assuming an underlying distribution of patient count between 1 and 10. Analyzing intervals as ordinal predictors (i.e., 1–10, 11–20, 21–30) did not meaningfully change results.

## Results

Among 725,169 MD or DO physicians with Medicare prescribing data, 341,644 (47.1%) had documented payments in the OPP data. Among those receiving payments, mean payment was $1750 (SD $28336); median was $138 (IQR $48-$394). Such payments were markedly right-skewed (S2 Fig).

**Fig 1:**
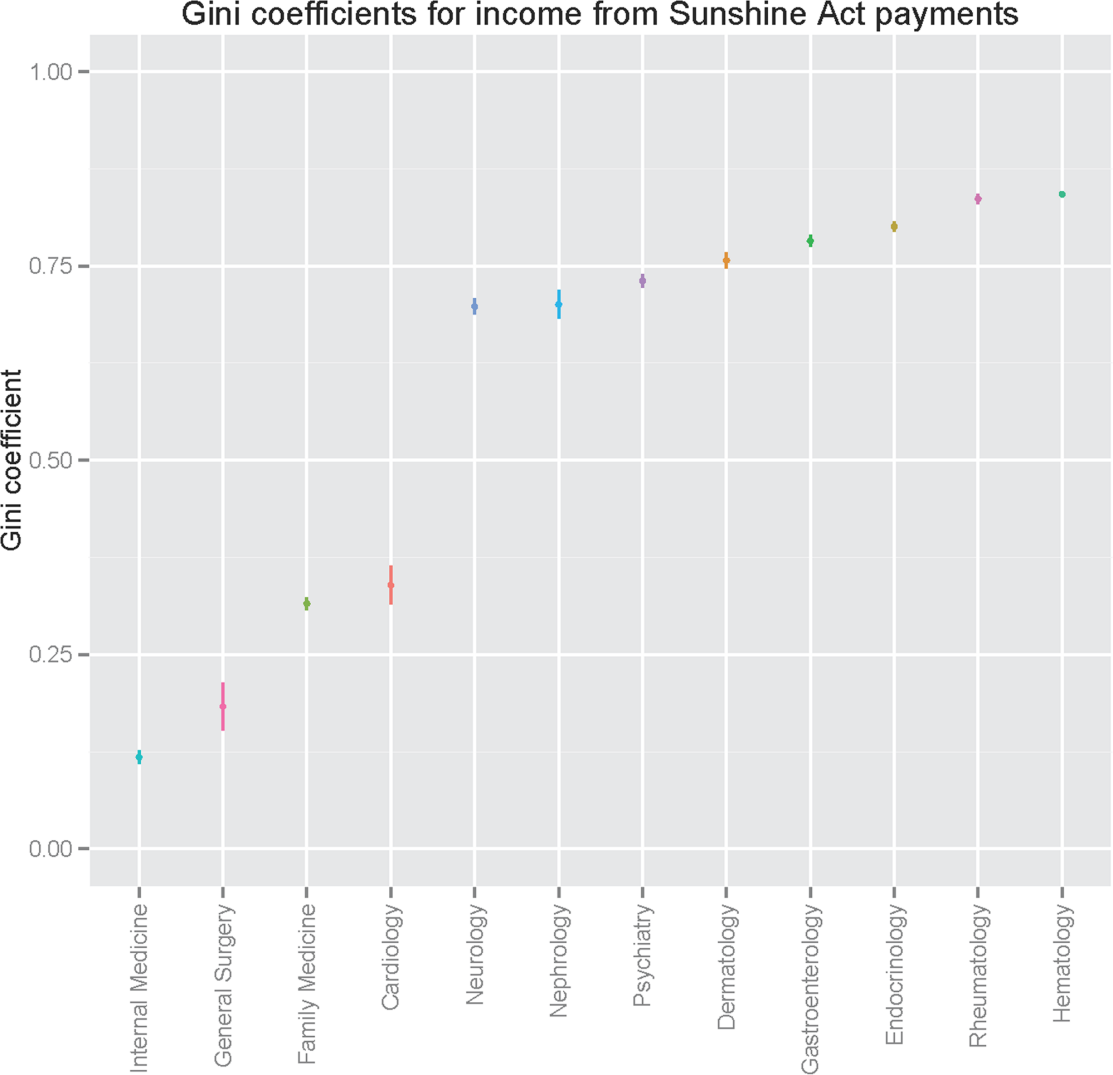
Gini coefficients for Sunshine Act payments, by specialty.

We first examined distribution of payments among a subset of specialties with more than 4,000 physicians represented in prescribing data. Fig 1 illustrates Gini coefficients by specialty, a measure of income distribution in a population, where 0 represents equivalent income for all individuals and 1 represents all payments accruing to a single individual. We identified marked differences in distribution between specialties; these ranged from 0.08 for family medicine to 0.83 for hematology/oncology (S1A Table).

Subsequent analyses considered income quintiles, compared to no income, within each specialty; specific ranges in each quintile by specialty are listed in S1B Table. Features of individuals in the top quintile of payment recipients, contrasted with all others, are described in Table 1. Features associated with being among the top quintile of payments within a specialty (p<0.001) included being male, having larger Medicare Part D patient panels, practicing in the South, and practicing in a higher-income zip code.

**Table 1:** Characteristics of physicians among the top quintile for receipt of payments in Q3/4 2013*.

	All Others			Top Payment Quintile		
	(N = 346,074)			(N = 39,836)		
**Feature**	**n**		**pct**	**n**		**pct**
Sex (female)	118,474		34.2%	7,749		19.5%
Region						
Midwest	78,065		22.6%	7,563		19.0%
Northeast	77,611		22.4%	8,799		22.1%
South	110,276		31.9%	15,619		39.2%
West	73,048		21.1%	7,337		18.4%
Other	7,074		2.0%	518		1.3%
Income tertile (by zip)						
First	102,954		29.8%	10,917		27.4%
Second	90,426		26.1%	9,975		25.0%
Third	141,264		40.8%	18,030		45.3%
(none)	11,430		3.3%	914		2.3%
Physician type (MD)	314,067		90.8%	35,935		90.2%
Fewer than 11 patients	34,863		10.1%	1,061		2.7%
**Feature**	**n**	**mean**	**SD**	**n**	**mean**	**SD**
Total payments received	346,074	$83.11	$201.34	39,836	$6,341.64	$29,474.29
Total drug cost	346,074	$172,723.30	$274,598.60	39,836	$454,154.40	$567,649.10
Patient count^	311,211	199	206	38,775	316	285
Total claims	346,074	2,428	4089	39,836	5,217	6,810
Branded claims^#^	210,584	757	1119	29,604	1,629	2,120
Generic claims^#^	300,921	2,156	3308	37,465	4,087	5,072

*All contrasts significant at p<0.001

^Patient counts only calculable for physicians with 11 or more patients; counts of individuals with fewer than 11 MPD patients shown above

^#^Claim counts only available for 11 or more claims

We next examined prescribing characteristics among these individuals; analyses were done within each major specialty. We utilized linear regression to examine association between prescribing cost per patient and payment quintile (or no-payment), adjusting for the practice- and physician-level features summarized in Table 1.

**Fig 2:**
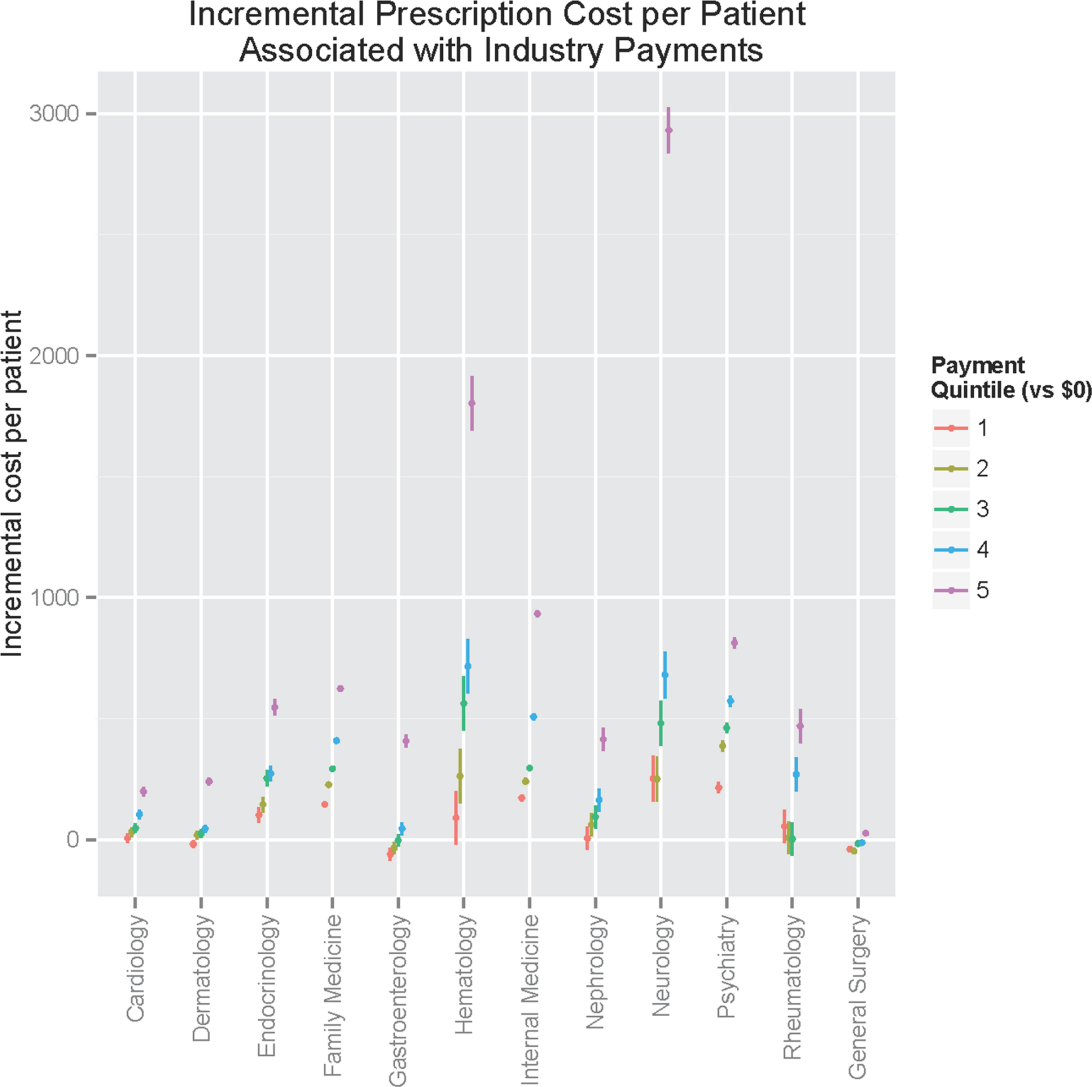
Incremental prescribing cost per patient, by payment quintile and specialty.

Adjusted associations are illustrated in Fig 2; by test for contrast of marginal linear predictions, clear linear dose-response relationships were identified for all specialties (p<0.0001 for test for linear trend across categories, except p = 0.0006 for general surgery; S2 Table). Greatest incremental prescribing cost compared to the physicians receiving no payment was observed among neurologists followed by hematologist/oncologists. (Similar relationships were observed when models were re-fit using only patients age 65 or greater and only patients younger than 65; likewise, estimates were stable when panel size was imputed for physicians with 10 or fewer MPD patient panels—see S3 Table). In sensitivity analysis, we incorporated median household income by zip code and number of patients receiving low-income supplement to Medicare Part D, two proxies for patient socioeconomic status; again results did not change meaningfully–S3 Table. Adjusting for total claim count—i.e., considering whether costs might be increased simply because of greater prescribing per patient—also yielded qualitatively similar results (S4 Table).

**Fig 3:**
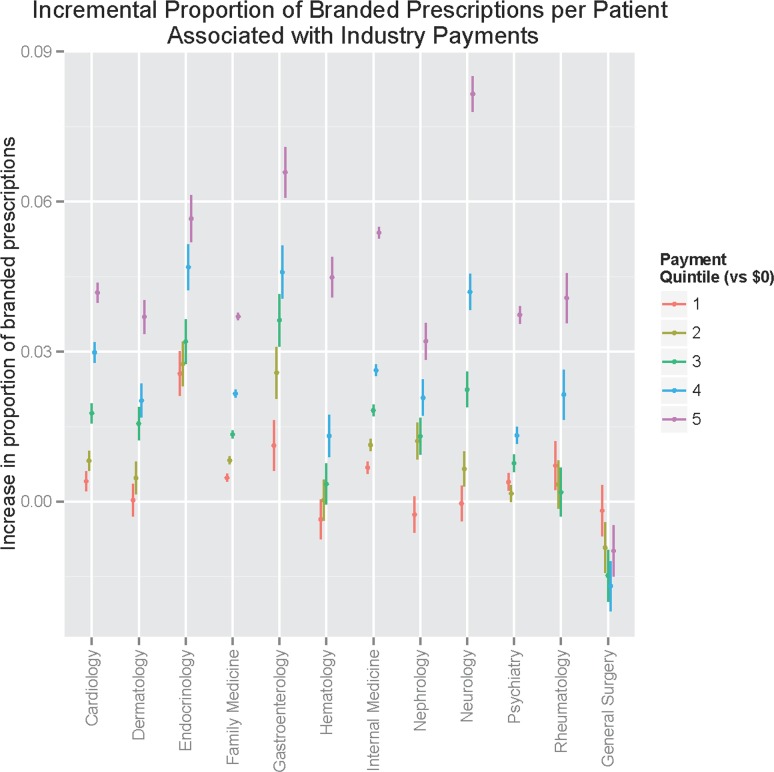
Incremental proportion of branded prescriptions, by payment quintile and specialty.

To understand the association between payments and brand versus generic prescribing, we repeated these regression analyses with proportion of branded prescriptions as the dependent variable (Fig 3); once again the top quintile of neurology exhibited the greatest increase in branded prescriptions, followed by gastroenterology and then endocrinology. Here too, general surgery did not exhibit a dose-response, and in fact receipt of payments was associated with prescribing a significantly lower proportion of branded medications.

**Fig 4:**
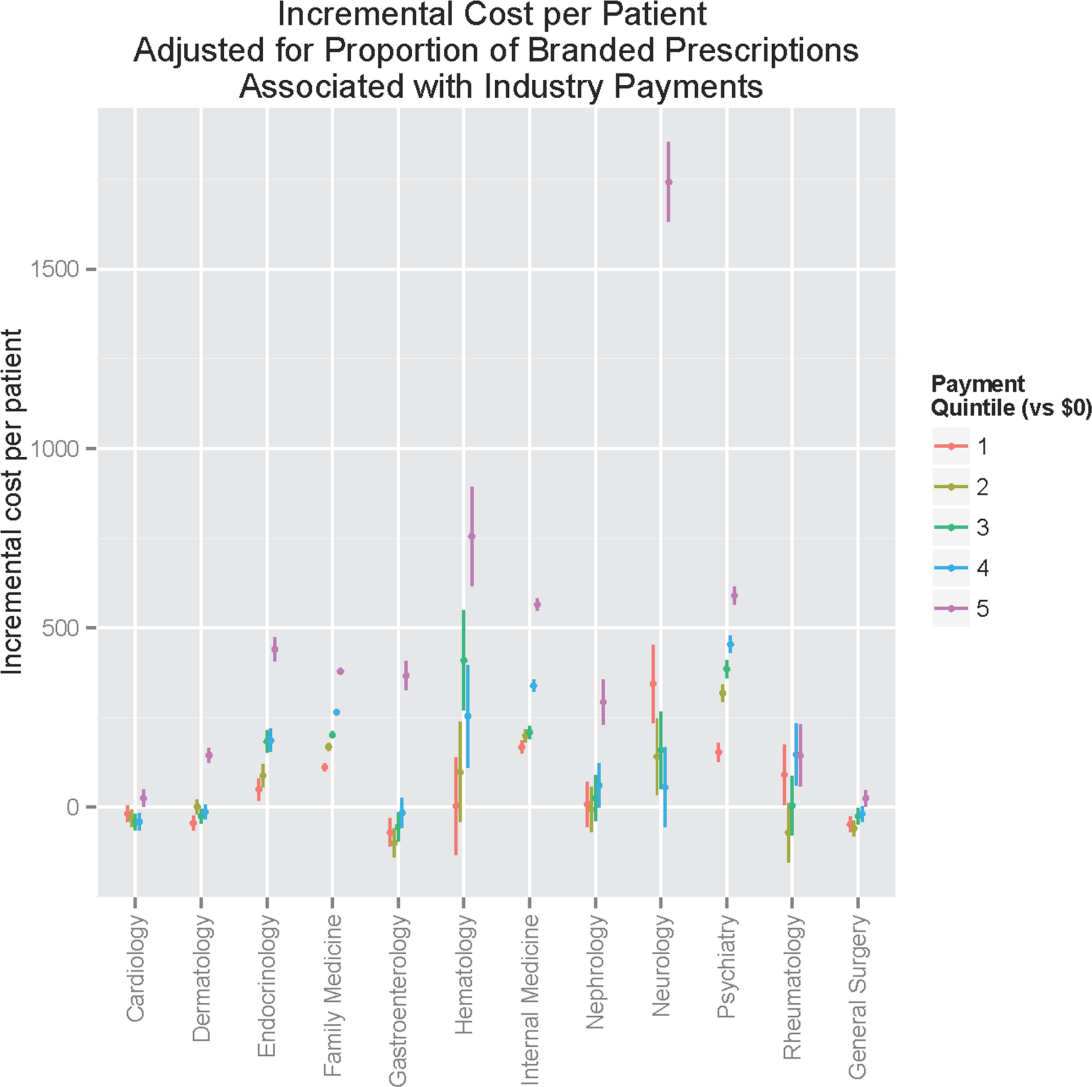
Incremental prescribing cost per patient, adjusted for proportion of branded prescriptions, by payment quintile and specialty.

Lastly, we repeated incremental cost analysis adjusting for proportion of branded prescriptions (Fig 4). This analysis allows an estimate of the extent to which the incremental cost observed can be explained by increased brand-name medication prescribing. Here, greater variation between specialties was observed. The previously-observed dose response persisted for most specialties, indicating that the proportion of branded prescriptions does not fully explain incremental cost. However, for others—particularly cardiology and rheumatology—no such relationship was observed, indicating that the previously observed associations were better observed by the greater proportion of branded prescriptions.

## Discussion

In this analysis of 12 months of prescribing data from more than 700,000 U.S. physicians, including analysis of nearly 400,000 individuals within 12 specialties, we find that receipt of industry payments is associated with greater prescription cost per beneficiary. These effects persist after adjustment for prescriber-level features including location, sex, and Medicare Part D panel size. In general, our results are consistent with a host of prior, substantially smaller investigations suggesting that exposure to pharmaceutical company information increases rates of prescribing,[5] extending these results in a very large physician cohort and across a range of clinical specialties and controlling for differences among practices. While Medicare Part D does reflect a select subset of overall patient populations (~72% of eligible individuals with Medicare had Part D coverage in 2015[13]), sensitivity analysis using patient subgroups suggested that these effects are not limited to older or younger patients. Further, despite wide variation in the amount of industry payment by specialty group, the relationship between greater payments and greater expenditure is quite consistent except among general surgeons.

On the other hand, differences emerge between individual specialties as well. Most notable is the variation in distribution of payments. In general, based on Gini score primary care-oriented specialties appear to have more even payment distributions, while some others—neurology, endocrinology—appear to be more highly concentrated among a smaller number of physicians. One reasonable explanation for this variation is heterogeneity of practice type and extent of specialization: some practices may treat primarily individuals with a particular set of disorders (e.g., autoimmune) for which costly treatments such as biologics have become available. In particular, this may help to explain the particularly large incremental costs associated with neurology, for example. It is also possible that the impact of highly-paid consultants or key opinion leaders such as those represented in the highest payment quintile is different across specialties. As we did not include research reimbursement or equity, however, these differences cannot be attributed directly to greater research involvement.

Interestingly, we observe two sorts of relationships between payments and prescribing pattern. For some specialties, increased cost per patient is driven primarily by more expensive medication prescribing overall. For others, however, cost is driven by a greater proportion of branded prescriptions. (Even in this latter category, however, the top quintile of payments is associated with significantly greater costs overall). In other words, we see evidence that physician payments are associated with more expensive medications and a shift to branded medications, but the relative contribution of each to overall cost varies by specialty.

Despite our effort to incorporate the limited data available to us about practice differences, we cannot fully address confounding factors—in particular, unobserved differences in practice types. For example, individuals with more academic affiliation may use newer and thus more expensive interventions, and be more available for industry relationships. Those who are more involved in medical education programs may be prone to use newer medicines (given industry sponsorship of medical education efforts), and to receive payments from industry sponsors. The associations may also be driven by a particular practice subgroup more likely to receive industry sponsorship, and to prescribe newer branded medications. Moreover, the fact that prescription data spans an entire year, while payment data only the last two quarters, precludes a strong claim about causation even if one assumes that payments were unlikely to change substantially from the first to the second half of the year.

Nonetheless, our observation of the association between physician payments and prescribing patterns indicate at least the possibility of a causal relationship in some circumstances. That is, the physicians with greater involvement with industry are more apt to prescribe branded and/or more costly medications. The distinct associations with cost—either greater overall cost per patient, or greater proportion of branded prescriptions—suggests that strategies to address these differential costs may depend on the specialty.

Our results may contribute to ongoing debate about the importance and usefulness of financial disclosure. While there is broad consensus about the importance of transparency in reporting financial conflicts of interest, there is a lack of clarity about how such reporting may be useful. For example, a public interest journalism site notes that patients should ask clinicians who receive payments whether there are less costly treatment options available.[14] While our results indicate that clinicians who receive greater industry payments may on average be more likely to prescribe branded treatments, it would seem a better way to assess this propensity is directly from the prescribing data. That is, rather than examining physician payments, patients might be better served simply examining summaries of prescribing patterns. Indeed, it has been argued that reporting simply shifts the burden of using such data to patients who may not be well-positioned to interpret these data. For example, one study showed patients may be more rather than less likely to enroll in clinical trials where the principal investigator has disclosed a conflict of interest.[15]

The impact of disclosure on clinicians themselves also bears consideration. In other fields with similar asymmetric knowledge relationships—financial advising, for example—disclosure may lead to more self-interested behavior on the part of advisors.[16, 17] Given this complexity, the need for additional empirical data to understand how these relationships impact accepted quality metrics—not just cost or prescribing—becomes important. Finally, our results are relevant for other stakeholders as well, most notably public and private health insurers. In an effort to limit the growth of pharmaceutical expenditures, many insurers have implemented funding and pricing policies to promote the use of less costly medicines. The present study indicates that payments to physicians may encourage the opposite behavior, such that limiting payments may represent another opportunity to control prescribing costs. By one estimate, costs of detailing to physicians by industry in the U.S. exceeded $20B, of which only a fraction likely represents direct physician payment[18].

We note several key limitations in these data. First, the data collection process has been widely criticized [19] as a result of inconsistencies in reporting requirements and the difficulty in identifying and correcting errors.[20] Second, the available prescribing data relates only to Medicare Part D benefits; it will be important to examine the associations we identify in other prescribing data sets to ensure they are not limited to Medicare providers, although these represent more than 90% of all non-pediatric physicians.[21] We also cannot exclude bias introduced by inability to match a small subset of clinicians—these may represent clinicians who do not treat Medicare patients, or those for whom identifying data is simply insufficient to reliably identify them. If anything, this misclassification should bias us towards the null—i.e., it should contribute to an underestimate of effect. In simulations under very conservative assumptions (i.e., that all unmatched individuals in a given specialty would have been at the 75%ile for prescribing costs), evidence of dose-response persists. Strangely, the same act that established the Open Payment Program requires collection of the provider identifiers needed for precise matching and expressly forbids their release by the federal government.

Our results underscore the striking absence of comparable large-scale Medicare quality data that would allow a critical question to be answered: does the additional expenditure by clinicians receiving payments correlate with changes in outcomes? While such quality metrics are challenging and likely to be highly disease- or specialty-specific, they are a necessary prerequisite for moving beyond purely descriptive or broad association studies using large-scale pharmacy data.

Nonetheless, taken together our findings indicate strong evidence of association between physician payments from industry and their prescribing patterns across specialties, effects which cannot be fully explained by practice size, proportion of older or low-income patients, physician sex, or geography. In an era of increasing efforts toward cost containment, understanding the extent to which monitoring or addressing these payments, particularly to those in the highest quintile of receipts, may help to constrain costs represents a critical next step.

## Supporting Information

S1 Fig CONSORT-like diagram of patient matching. (PDF)

S2 Fig Distribution of Sunshine Act payments, by specialty. (PDF)

S1 Table Gini coefficients for (a) and quintiles of (b) Sunshine Act payments, by specialty.
(XLSX)

S2 Table Incremental prescribing cost per patient, evidence for linear trend. (XLSX)

S3 Table Incremental prescribing cost per patient, by payment quintile and specialty: sensitivity analysis. (XLSX)

S4 Table Incremental prescribing cost per patient, by payment quintile and specialty, adjusted for total claim count. (XLSX)
