# Supplementary material for: Physician Payments from Industry Are Associated with Greater Medicare Part D Prescribing Costs
Source: PLoS One. 2016 May 16;11(5):e0155474. doi: 10.1371/journal.pone.0155474 (PMC4868346; doi:10.1371/journal.pone.0155474)
Supplement: S1 Fig — (PDF) [file pone.0155474.s001.pdf]

Supplemental Figure. CONSORT-like diagram of matching process

Database cleaning

Records in 2013 Medicare Part D database (MPD):
N = 1,049,381

=> omit 6,755 non-individual records

n = 1,042,646 MPD individuals

=> omit 317,477 non-DO and non-MD individuals

n = 725,169 MPD individuals with DO or MD degree

Records in 2013 Open Payment Program database (OPP):
N = 4,055,634

=> omit 23,247 non-individual records

n = 4,032,387 records (multiple records per individual)
= 468,974 OPP individuals

=> omit 61,754 non-DO and non-MD individuals

n = 407,220 OPP individuals with DO or MD degree

Physician matching:

Round 1: first/last/middle/zip	n=295,541	85.38%
Round 2: first/last/zip	n=20,402	5.89%
Round 3: first/last/middle	n=30,191	8.72%

=> omit 61,086 unmatched individuals in OPP

n =346,134 matches remaining

=> omit 3,872 ambiguous from wave 3

n =342,262 matches remaining

=> collapse 618 duplicate NPI

*n =341,644 matched individuals with MD or DO degree^*

^ 65,576 (16.1%) unmatched MD or DO individuals in MPD data after exclusion of incorrect/ambiguous matches and collapse of duplicates.
