# Supplementary figures and images for: Physician Payments from Industry Are Associated with Greater Medicare Part D Prescribing Costs
Source: PLoS One. 2016 May 16;11(5):e0155474. doi: 10.1371/journal.pone.0155474 (PMC4868346; doi:10.1371/journal.pone.0155474)

Distribution of income from Sunshine Act payments

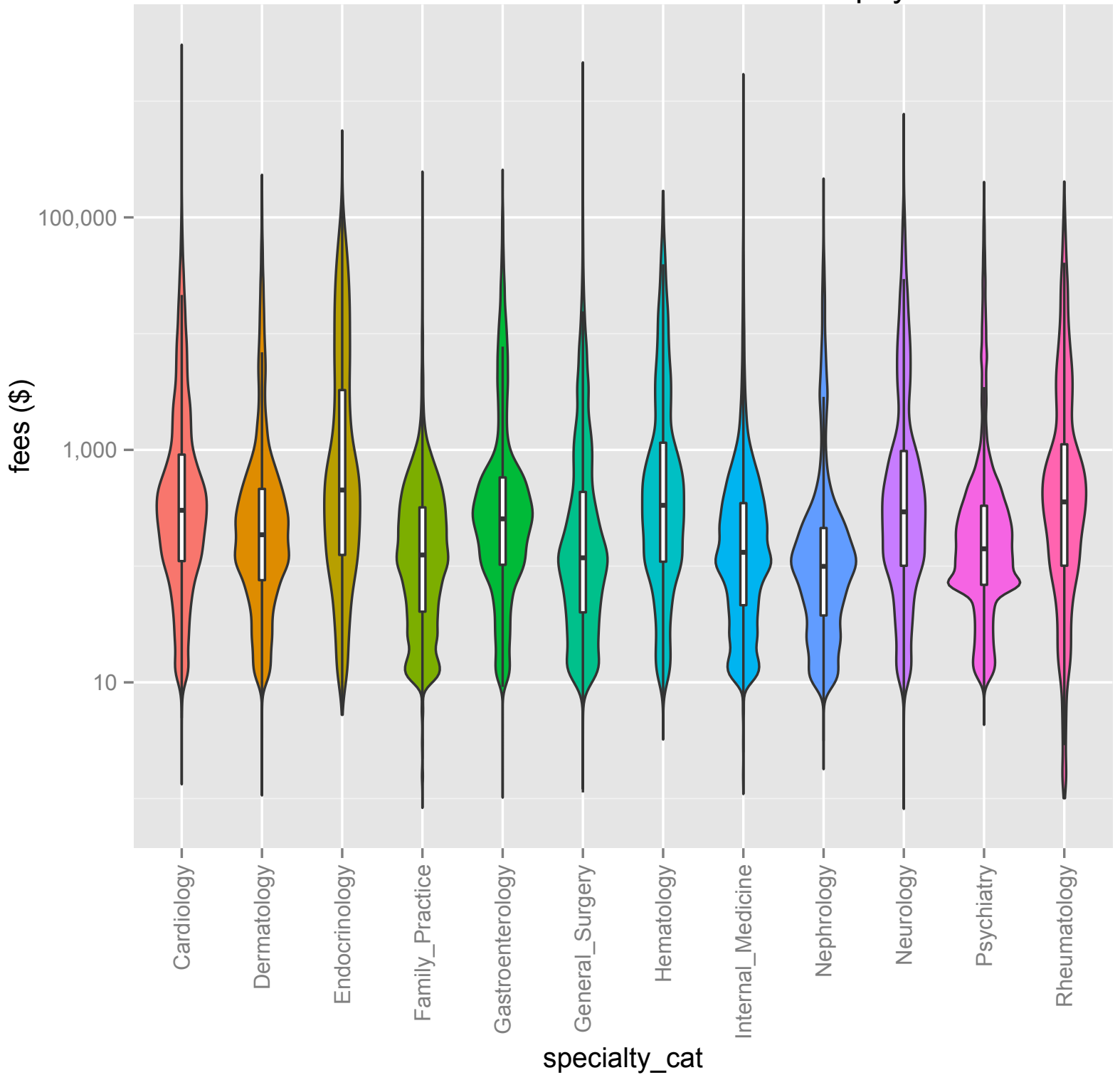

Supplement: S2 Fig — (PDF) [file pone.0155474.s002.pdf]
